# Development of a conceptual model of the capacity for patients to engage in their health care: a group concept mapping study

**DOI:** 10.1186/s12913-023-09785-x

**Published:** 2023-08-10

**Authors:** Gennaro Di Tosto, Jennifer L. Hefner, Daniel M. Walker, Megan E. Gregory, Ann Scheck McAlearney, Cynthia J. Sieck

**Affiliations:** 1 CATALYST, Center for the Advancement of Team Science, Analytics, and Systems Thinking, College of Medicine, The Ohio State University, 700 Ackerman Rd, Suite 4100, Columbus, OH USA; 2 Department of Family and Community Medicine, College of Medicine, The Ohio State University, Columbus, OH USA; 3 Division of Health Services Management and Policy, College of Public Health, The Ohio State University, Columbus, OH USA; 4 Department of Health Outcomes and Biomedical Informatics, College of Medicine, University of Florida, Gainesville, FL USA; 5 Center for Health Equity, Dayton Children’s Hospital, Dayton, OH USA

**Keywords:** Patient engagement, Patient-centered care, Group concept mapping.

## Abstract

**Background:**

Patient engagement is seen as a necessary component in achieving the triple aim of improved population health, improved experience of care, and lower per capita health care costs. While there has been a substantial increase in the number of tools and patient-centered initiatives designed to help patients participate in health decisions, there remains a limited understanding of engagement from the perspective of patients and a lack of measures designed to capture the multi-faceted nature of the concept.

**Methods:**

Development of a concept map of patient engagement followed a five-step modified Group Concept Mapping (GCM) methodology of preparation, generation, structuring, analysis and interpretation. We engaged a Project Advisory Committee at each step, along with three rounds of survey collection from clinicians and patients for element generation (272 clinicians, 61 patients), statement sorting (30 clinicians, 15 patients), and ranking and rating of statements (159 clinicians, 67 patients). The survey of three separate samples, as opposed to focus groups of ‘experts,’ was an intentional decision to gain a broad perspective about the concept of patient engagement. We conducted the structure and analysis steps within the groupwisdom concept mapping software.

**Results:**

The final concept map comprised 47 elements organized into 5 clusters: Relationship with Provider, Patient Attitudes and Behaviors, Access, Internal Resources and External Resources. There was considerable agreement in the way elements in each cluster were rated by patients and clinicians. An analysis of the importance of the constitutive elements of patient engagement relative to their addressability highlighted actionable items in the domain of Relationship with Provider, aimed at building trust and enabling patients to ask questions. At the same time, the analysis also identified elements traditionally considered barriers to engagement, like personal access to the internet and the patient’s level of digital literacy, as difficult to address by the healthcare system, but also relatively less important for patients.

**Conclusions:**

Through our GCM approach, incorporating perspectives of both patients and clinicians, we identified items that can be used to assess patient engagement efforts by healthcare systems. As a result, our study offers specific insight into areas that can be targeted for intervention by healthcare systems to improve patient engagement.

**Supplementary Information:**

The online version contains supplementary material available at 10.1186/s12913-023-09785-x.

## Background

Patient engagement (PE) has been called the next “blockbuster drug” due to its potential to improve health outcomes and possibly generate significant healthcare savings [1]. With increasing emphasis on engaging patients as partners in and often drivers of their health and health care [2], PE is seen as a necessary component in achieving the triple aim of improved experience of care, improved health of populations, and lower per capita health care costs [1, 3]. Healthcare systems have responded to this emphasis in a rapid and pervasive manner, and policy and organizational efforts have advanced the concept of truly patient-centered care [4]. Further, the number and variety of tools that help patients participate in health decisions, as well as the introduction of patient-centered initiatives to encourage engagement, have increased substantially.

Yet, despite high expectations for patient engagement, there remains a limited understanding of engagement from the perspectives of patients and a lack of measures designed to capture the concept of engagement [5]. These deficits make it difficult for healthcare systems to effectively support PE [6]. PE has been defined in a number of ways: (1) patients’ involvement in care and decision making; (2) the actions patients take to obtain benefits from health care services; (3) the thoughts, feelings, and actions that are present at different stages of PE; or, (4) the specific behaviors that patients can take to be engaged in their health and health care [7–9]. Alternatively, PE has been conceptualized as multifaceted and inclusive of elements of each of these definitions. For example, the first phase of the Interactive Care Model for engaging patients in care is to assess the person’s capacity for engagement before discussing choices and making plans [10]. The authors note that accomplishing the first phase is particularly challenging because many factors can influence engagement capacity. This complexity was also identified in a systematic review of PE interventions studies that found more than 20 different variables were used to assess capacity for engagement with formal measures only developed for a subset of these factors [11].

Recognizing the complexities of defining and measuring PE, we engaged in a modified group concept mapping (GCM) process. GCM is a flexible yet rigorous approach to clarifying and elucidating complex concepts, and has been described as an inclusive, participatory, collaborative, and inductive social science process [12, 13]. By engaging stakeholders in concept map development, researchers can gather a range of perspectives on elements that comprise a concept, and collaboratively develop an intuitive representation of relationships within the concept. This method has been applied across a range of disciplines for developing new measures [14, 15], frameworks [16], and intervention designs [17].

Our concept map fills a gap in both research and practice by explicating the different aspects underlying PE, in order to support efforts that can be prioritized by healthcare systems to increase PE and patient-centered care. To date, few studies have utilized concept mapping to address patient engagement. One of our primary contributions is our inclusion of patients in this process, a point which has thus far seen limited yet promising applications (see, e.g., the work of Ogden and colleagues [18]). The specific goal of this study was to develop a conceptual model of patient engagement that reflects the shared perspectives of patients and providers about engagement and takes into account the context in which engagement occurs. Additionally, to provide guidance to healthcare systems in focusing their PE efforts, we examined the perspectives of physicians and patients about the importance and addressability of each element of PE.

## Methods

Development of the concept map entailed a modified GCM process, consisting of the five traditional steps of preparation, generation, structuring, analysis, and interpretation [19–21]. We describe the process as modified because we used survey methods for the generation and structuring steps, instead of the more traditional approach of conducting focus groups with experts. This modification was an intentional choice due to the nature of our research question. Rather than employ a small group of patient engagement ‘experts’ to brainstorm the elements of PE, we felt this topic required the input of the true patient engagement stakeholders — the patients. Both clinicians and patients from across the U.S. completed our surveys, providing diverse perspectives on PE. Table 1 presents the five steps and shows the activities associated and participants involved with each step.

### Preparation

Preparation was an essential step to develop our recruitment protocols and finalize the survey prompts for idea generation in the next step. This step was accomplished with the Project Advisory Committee (PAC) of clinicians (n = 4), patient advisors (n = 7) and research team members (n = 5). A patient advisor is the term describing patients and caregivers who serve on ‘Patient Advisory Boards’ for a specific clinic or a healthcare organization. By definition, these advisors are also engaged personally with the healthcare system; therefore, we relied on them to incorporate patient perspectives into the GCM process.

The survey included 14 open-ended items asking respondents to share which elements they perceived as influencing engagement, barriers to engagement, and facilitators of engagement such as tools and skills. Participants were also asked to provide a description of an engaged patient. Questions included: “What concepts come to mind when you hear the term patient engagement?” “What do patients who are engaged in their healthcare do?” “How do patients who are engaged feel and/or believe?” “What do patients who are engaged think and/or know?” “What types of tools and technology do you think patients need to fully engage them in their healthcare?” “What skills do patients need to have to engage in their healthcare?” “What other things do patients need to fully engage in their healthcare?”

**Table 1:** Modified Group Concept Mapping (GCM) steps, activities and participants

GCM steps	Activity	Participants
Preparation	Develop recruitment protocols	Project Advisory Committee
	Finalize survey prompts for idea generation	Project Advisory Committee
Generation	Field an online survey of open-ended questions	727 clinicians and 61 patients
	Assess sufficiency of responses	Research team
	Code write-in responses using a codebook	Research team
	Generate a list of items for structure steps	Research team
Structure	Sort items using groupwisdom software	30 clinicians and 15 patients
	Online survey to rank and rate items	269 clinicians and 103 patients
Analysis	Multi-dimensional scaling and Cluster analysis	groupwisdom software
	Review point maps and proposed clusters	Research team using software output
	Identify ideal cluster map and name clusters	Project Advisory Committee
Interpretation	Draft a Concept Map based on the selected clustering solution	Project Advisory Committee

Note: Project Advisory Committee included clinicians (n = 4), patient advisors (n = 7) and research team members (n = 5)

### Generation

In the *generation* step, we administered an online survey to clinicians and patient advisors across the U.S. recruited through institutional and professional association listservs between May and November 2019. Upon survey completion, participants received a $20 gift card in appreciation of their time.

We analyzed the open-ended responses using a thematic analysis approach [22], in which 4 coders on the research team developed a preliminary codebook based on the survey prompts, then coded the first 40 responses to each question, modifying the codebook as needed and discussing differences to reach consensus. Once consensus was reached, the remaining responses were divided among 4 research team members who coded individually and met frequently to discuss concerns and adapt the codebook. The research team used Atlas.ti v8 to support the coding process [23]. The outcome of this activity was a list of items related to patient engagement, as identified by clinicians and patients, to be used in the structure step.

### Structure

The *structure* step included two substeps: sorting and rating. First, participants used Concept System’s groupwisdom software [24] to sort the elements from the generation step into categories according to their perceived commonality. The sample for this step included a new group of clinicians and patient advisors from across the U.S. recruited through institutional and professional association listservs from October 2020 to January 2021. All elements in this step were labeled with their code label, and participants were given code definitions to ensure uniform understanding. Participants could create as many categories as they felt necessary but were required to include at least two items in each category. They also had the option to name each category. For the rating substep, from May-July 2021, we conducted a separate online rating and ranking survey in which participants were asked to assess, on a scale from 1 to 10, the relative importance of each item for patient engagement (1 = less important; 10 = more important), the item addressability by the healthcare system (1 = less addressable; 10 = more addressable), and how much each item was addressable by the patients themselves (1 = less addressable; 10 = more addressable). This survey was conducted outside the groupwisdom software, using Qualtrics survey software [25] instead to allow us to customize the ranking and rating questions to our research question.

In this substep, participants were also asked to rank items, but many clinicians did not address that part of the survey, and in the majority of occasions, participants assigned the highest rank to all the items. Consequently, ranking data were not used for analysis due to missingness and lack of variance.

### Analysis

We then engaged in step three, *analysis*, utilizing groupwisdom to generate candidate point maps and concept maps. Point maps are a bi-dimensional representation of the sorted items resulting from multidimensional scaling. Concept maps are produced via hierarchical clustering with the goal to highlight the conceptual structure of the map by partitioning the items in their 2D representation and aggregating them into groups on the basis of their relative distance. This process was enhanced by the retrieval of the items’ bridging value calculated by groupwisdom: a measure ranging from 0 to 1, and indicating the strength of the relationship with its surrounding items. Lower values indicate an anchor, or an item with strong relationships to its location on the map; higher values denote bridges, or items with dispersed relationships with points elsewhere on the map. These relationships were visually inspected via an activity called spanning analysis, which supported conceptual interpretation by drawing weighted edges between items. We subsequently worked with our PAC to compare candidate maps with varying numbers of clusters to determine a solution that was perceived as most appropriate to the study domain. We sought consensus across the group to select the most appropriate map. Additionally, in collaboration with our PAC, we generated a name for each cluster (i.e., a cluster of statements about transportation and cost of services became the ‘external resources’ aspect of PE). This step took place in February 2021.

Item ratings provided by clinicians and patient advisors in the structure step were processed further to augment the interpretation of the concept map with two visualization techniques proposed by Kane and Trochim: ladder-graphs (i.e., pattern matches) and bivariate graphs partitioned into quadrants by the average values (i.e., go-zones) [21]. Respondents who did not provide any answers to the survey (94 out of 269 clinicians, or 35%, and 26 out of 103 patients, or 25%) were excluded from the sample. The analyzed sample comprised a total of 252 respondents; missing values were handled via pairwise deletion. Mean values were calculated for each individual statement as well as for clusters. Cluster-level means were assessed via pattern matches to visually compare the relative importance of each cluster with their addressability by the health system and by the patient. Mean ratings for individual elements were analyzed using go-zones within each cluster to identify items deemed simultaneously important and addressable, which can represent potentially important intervention opportunities.

We initially created separate visualizations for the ratings provided by clinicians and patient advisors; however, since they showed similar results, their responses were combined to facilitate a consensus assessment of the concept areas of patient engagement.

### Interpretation

In step five, we conducted *interpretation* with our PAC. Specifically, all members of the PAC met to discuss the draft concept map and the rating data. This step took place in a working meeting in which all the different concept map visualizations—the point map, the cluster map, the point rating map, and the cluster rating map—were shared with the PAC for discussion about the appropriateness of the names to assign to each cluster and the individual items rating activity. This meeting occurred in February 2022.

## Results

Across the steps, respondents included in the analysis were: 727 clinicians and 61 patients in the generation step, 30 clinicians and 15 patients in the structure step, sorting substep, within groupwisdom, and 175 clinicians and 77 patients in the structure step, rating substep. Clinicians were primarily physicians practicing in a variety of specialties with 1–10 years of experience; 44% represented other roles, including nurses (18.6%) and physician assistants (13%).

### Analysis products

**Fig. 1:**
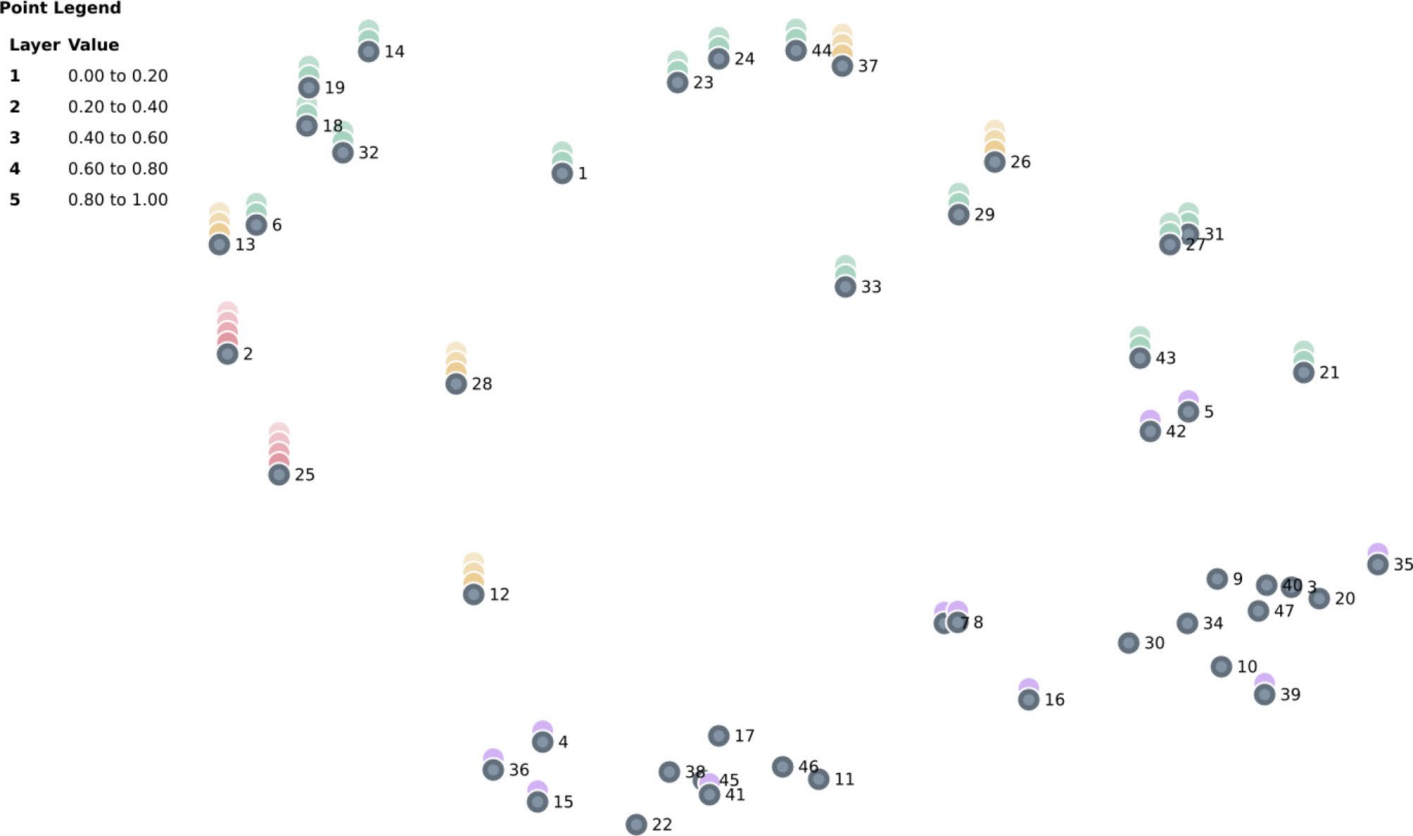
The 47 elements organized into a point map by groupwisdom, with color-coded layers representing bridging value quantiles. Lower layers indicate anchoring items; higher layers indicate bridging items

The multidimensional scaling analysis resulted in the point map depicted in Fig. 1. The 2-dimentional structure had a stress value of 0.185, which was considered good. According to meta-analytic studies reported by Kane and Trochim ([21], p. 98), typically stress values have an average of 0.285. In many GCM projects, stress has been reported in the range 0.10 to 0.35, with lower values being preferred ([13], p. 68).

### Concept map clusters

**Fig. 2:**
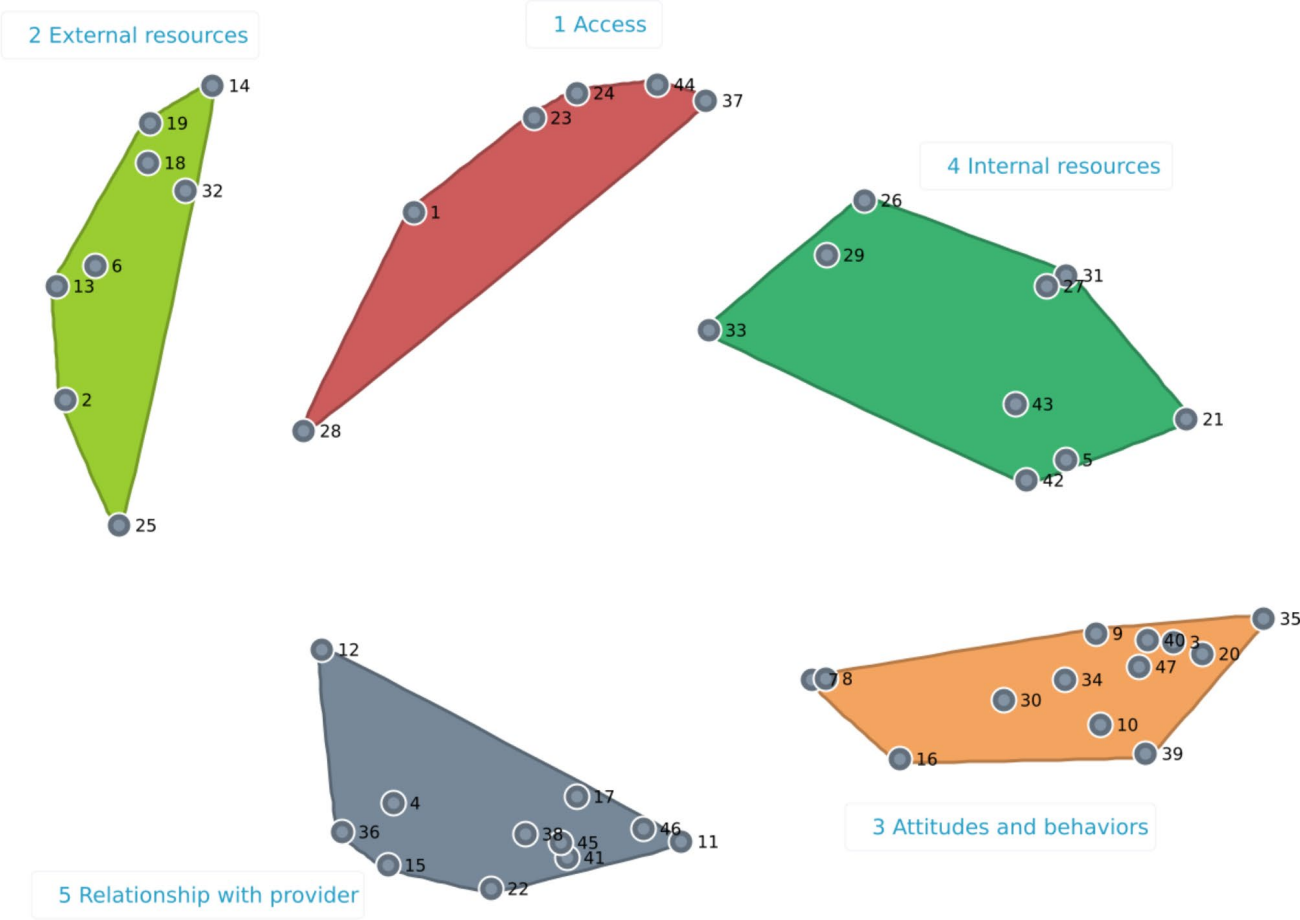
Concept map presenting 47 elements organized into 5 clusters labelled by the Project Advisory Committee

Our final concept map, shown in Fig. 2, organizes the 47 items into 5 clusters, with the names discussed and agreed upon by the PAC: cluster 1 “Access” (including transportation and cost of services), cluster 2 “External resources” (such as patient portals or educational materials), cluster 3 “Attitudes and behaviors” (including self-efficacy and resiliency), cluster 4 “Internal resources” (including literacy, understanding of the healthcare system, and support systems), and cluster 5 “Relationship with provider” (including trust and rapport). The full list, together with the bridging values calculated for each cluster, is available in the supplementary material (Additional file 1).

**Fig. 3:**
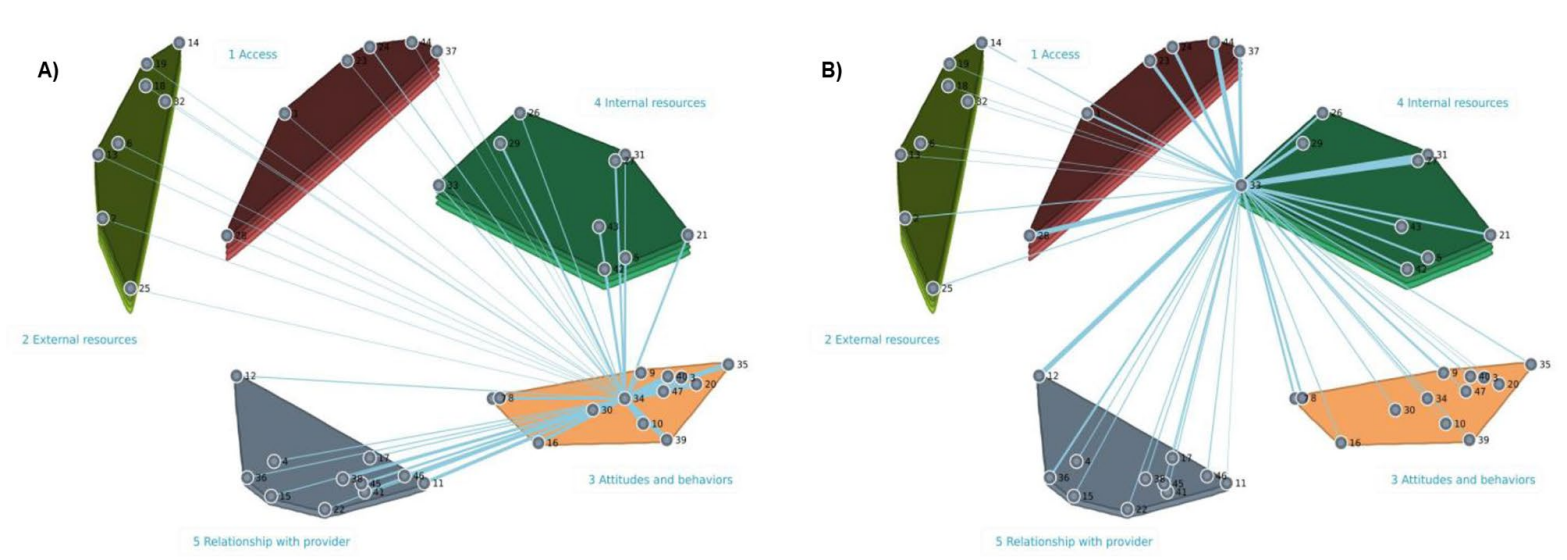
Spanning analysis. Examples of an anchoring item (**A**) and a bridging element (**B**)

Clusters 3 and 5 on the map of Fig. 2 were those with the lowest bridging values. Their items were more frequently sorted together and can be considered anchored: item 34 “Patient’s positive attitude”, for example (see Fig. 3.A), showed a bridging value of 0.059 and had strong ties with other items of the cluster number 3 “Attitudes and behaviors”, like “Resiliency” (item 39), “Patient comes to appointment prepared” (item 35), and “Patient asks questions” (item 30). Spanning analysis of the items contained in the other three clusters showed that many could be considered barriers or facilitators for items in other parts of the map. For example, item 33, “Patient’s language” (see Fig. 3.B), with a bridging value of 0.481, had many strong connections with items outside the “Internal resources” cluster (cluster 2): “Culturally appropriate care” (item 12 from cluster 5 “Relationship with provider”) and “Transportation” and “Medical jargon” (item 44 and 28, respectively, from cluster 1 “Access).

### Element rating, importance and addressability

**Fig. 4:**
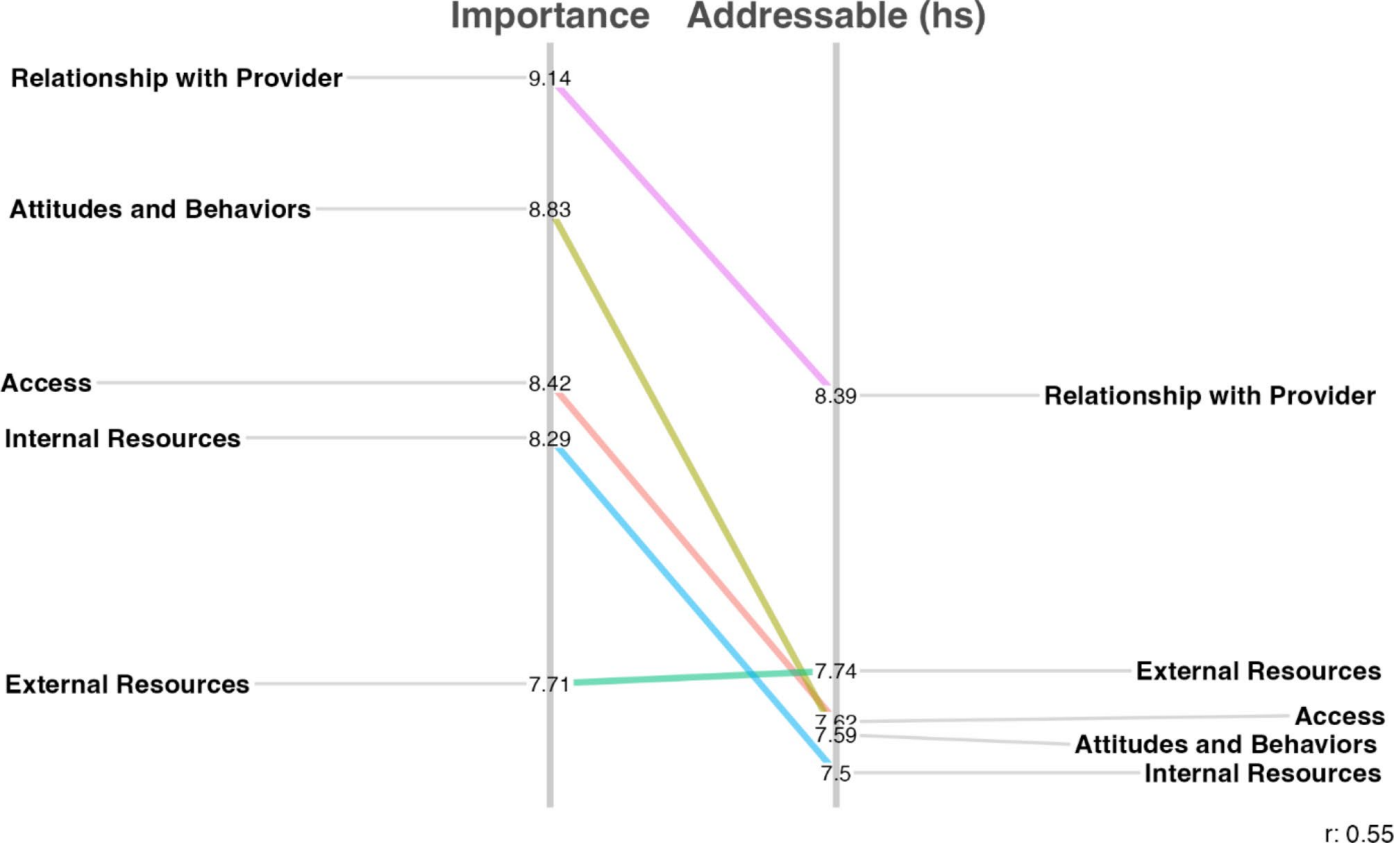
Pattern match comparing mean ratings of importance and addressability by the healthcare system at the cluster level

There was considerable agreement in the way items in each cluster were rated by clinicians and patients in the structure rating substep. Therefore, clinician and patient responses were grouped together for analysis, with the average ratings within each cluster presented using pattern matching. Figure 4 presents the relationship between the perceived item importance and their addressability by the healthcare system. All clusters were highly rated and perceived as important by the respondents, as indicated by the limited ranges of the scales. The average ratings were also positively correlated (r = 0.55), indicating that a high importance was associated with high addressability by the healthcare system. Overall, Relationship with Provider was deemed as the most important conceptual cluster, and the one that the healthcare system was most capable to address.

External Resources, while still averaging above the midpoint of the Likert scale, scored lowest for its importance while being the second most addressable aspect for the healthcare system. The remaining three clusters of the concept map—Attitudes and Behaviors, Access, Internal Resources—varied in the importance attributed to them but scored similarly as less addressable by the healthcare system.

**Fig. 5:**
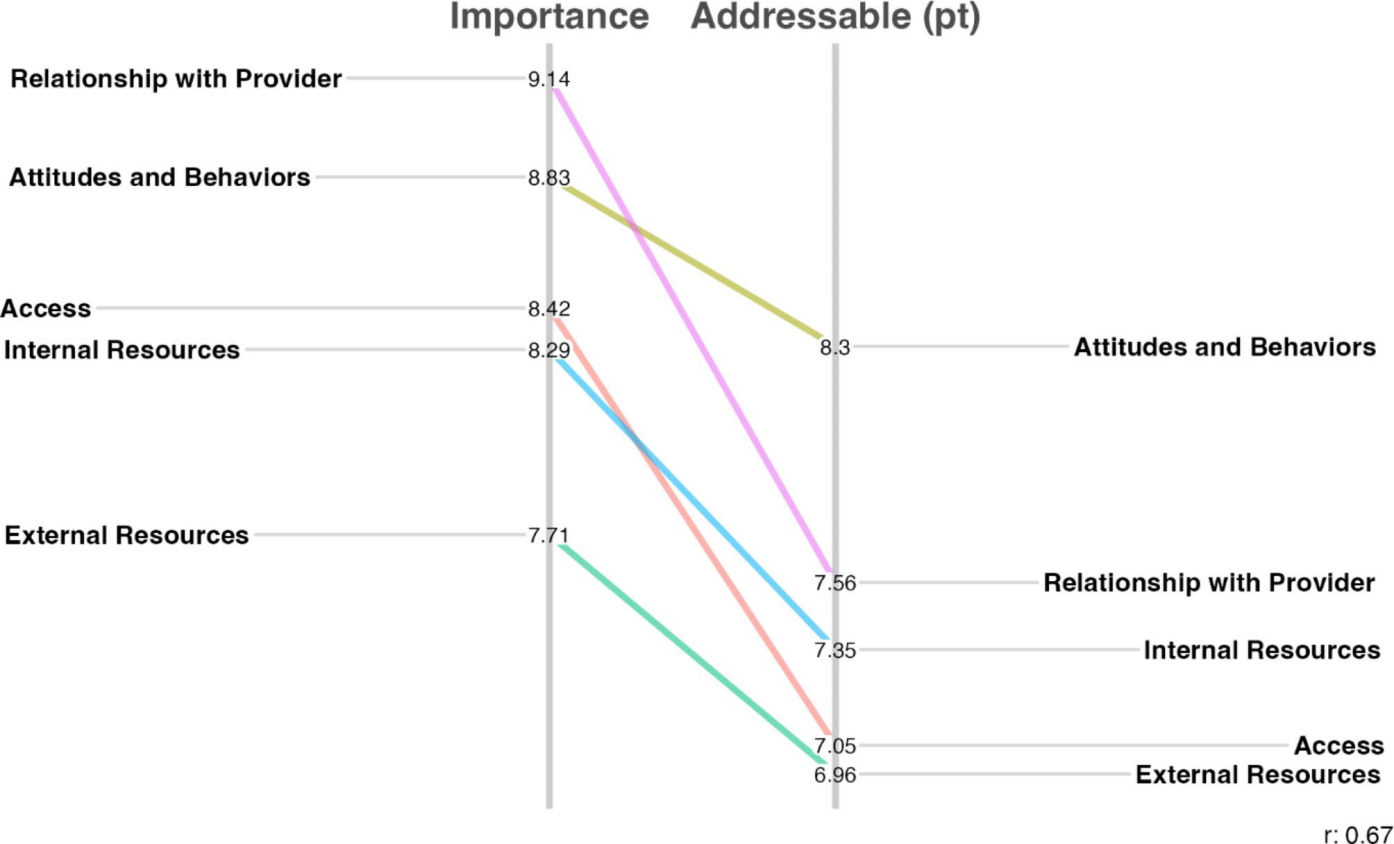
Pattern match comparing mean importance and addressability by patient ratings at cluster level

When focusing on addressability by the patients (see Fig. 5), we observed that clusters were once again highly rated and positively correlated (r = 0.67). External Resources shifted to become the least addressable cluster of the PE concept map, while Attitude and Behaviors was considered the most addressable cluster.

### Go-zones and actionable items

When looking at the individual items within a cluster, their relative assessments can help identify potential intervention points in the areas that are both important and addressable. Bivariate plots known as go-zones divide quadrants based on the mean values of importance and addressability, creating quadrants of differing sizes that suggest highly actionable elements.

#### Elements addressable by the healthcare system

**Fig. 6:**
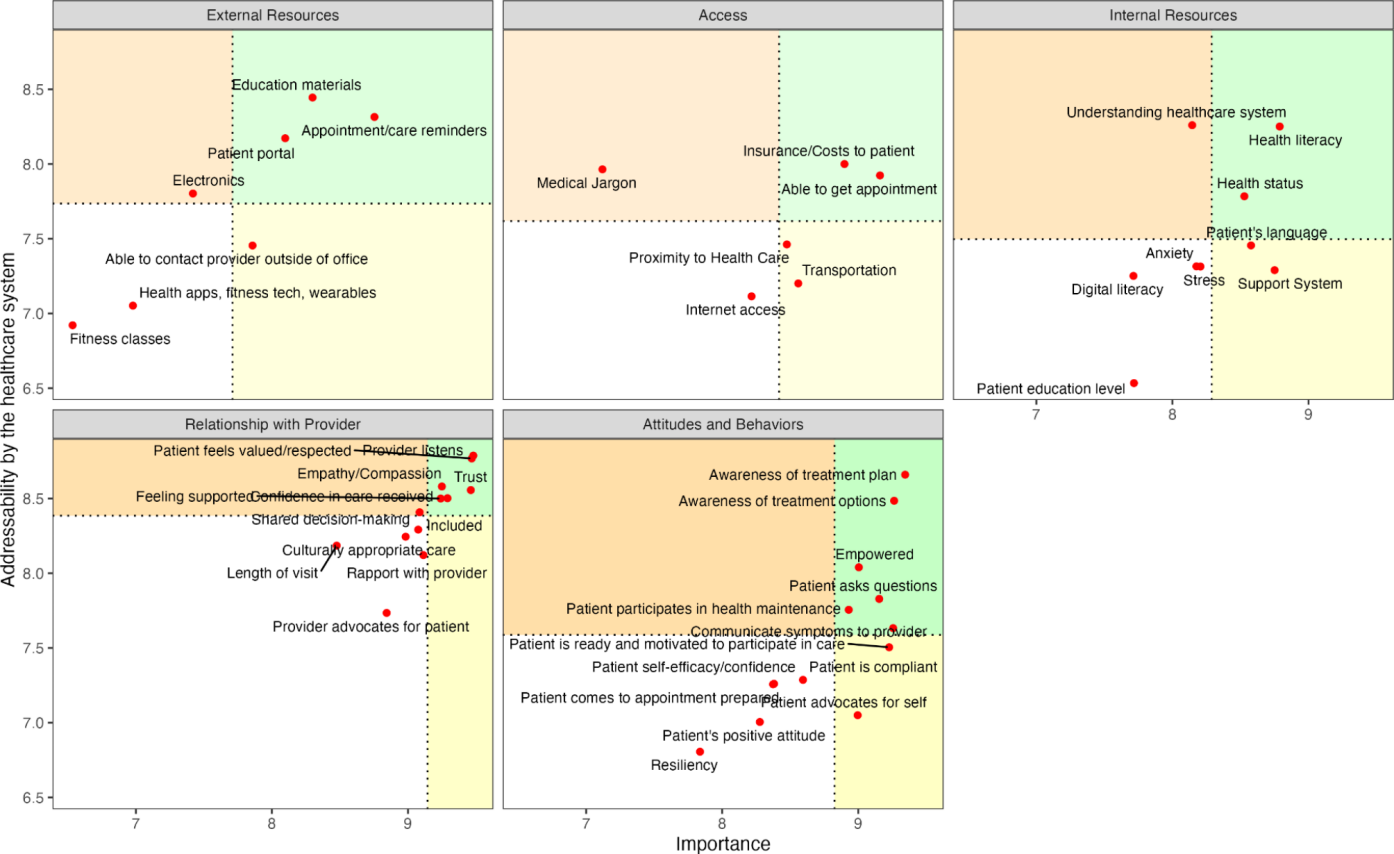
Item’ addressability by the healthcare system relative to their importance. Green areas indicate the go-zones relative to the five clusters of the patient engagement concept map, with dotted lines marking the average cluster ratings for importance and addressability, and red points marking the mean value of each element

The items characterizing a patient Relationship with their Provider were all rated very highly by the respondents, as reflected by the visual representation in Fig. 6. Even though all items are skewed towards the top-right corner, trust, compassion, and a feeling of confidence in the care received are all present in the go-zone highlighted in green.

The addressability of items composing the External Resources cluster appeared approximately linearly related to their perceived importance. Of those, patient portals, educational materials and appointment reminders were captured by the go-zone and appeared to be a consistent area of intervention.

Attitudes and Behaviors were viewed as relatively less addressable, despite being considered important elements of patient engagement. Among those, however, being aware of treatment options and treatment plans as well as asking questions and communicating about symptoms were four items based on information exchange that appeared in the cluster’s go-zone. Interestingly, patient’s resiliency, self-confidence and confidence were flagged as items less addressable and not as important compared to other items in the cluster.

The remaining two clusters, Access and Internal Resources, were identified as less actionable with items including access to the internet, digital literacy, patient educational level, and stress and anxiety. The ability to get appointments and factors influencing care cost for patients were instead actionable items captured by the go-zone as factors related to health literacy and health status.

**Fig. 7:**
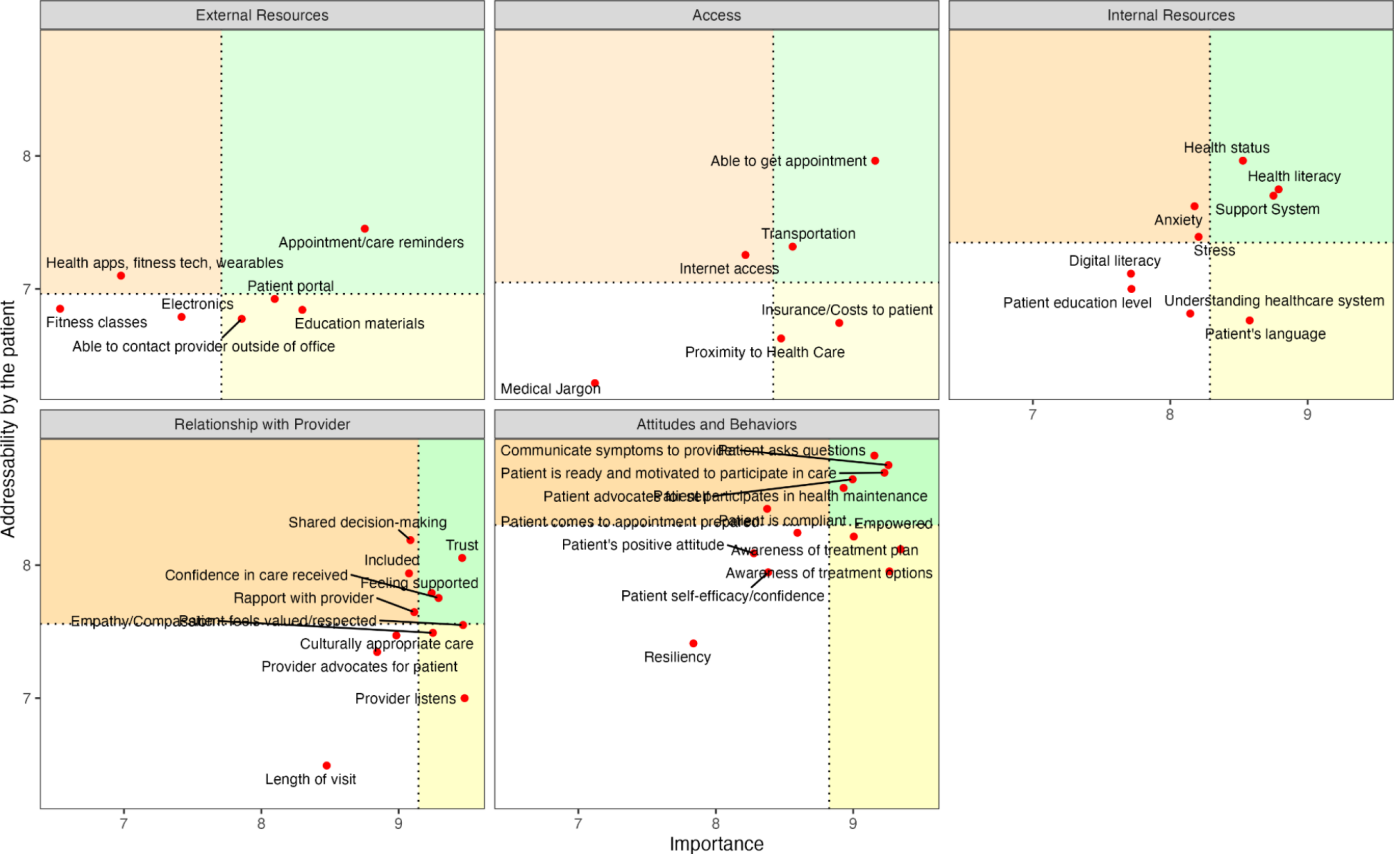
Items’ addressability by the patient relative to their importance. Green areas indicate the go-zones relative to the five clusters of the patient engagement concept map, with dotted lines marking the average cluster ratings for importance and addressability, and red points marking the mean value of each element

### Elements addressable by the patient

Figure 7 visually summarizes the go-zones related to the items’ addressability by the patient. Within Attitudes and Behaviors, the cluster rated most addressable by the patient, the items connected to asking questions, communicating symptoms, advocating for one’s self and participating in health maintenance were identified as both important and addressable. In the Relationship with Provider cluster, statements viewed as highly addressable by patients included trust in providers, feeling supported, confidence in care provided, and feeling respected.

Internal Resources was rated third most addressable by patients on average, with health status, health literacy, and availability of a support system identified in the go-zone. In the Access cluster, ability to get an appointment and transportation were the most important and addressable. Only one item in the External Resources cluster, appointment reminders, was rated both highly important and highly addressable by the patient.

## Discussion

Patient engagement is viewed with great optimism by healthcare providers with respect to how it can improve health outcomes, enhance patient satisfaction, and even lower costs. However, literature to date offers few measurement tools to assess patient engagement, and measures that do exist often lack conceptual grounding [26] and/or incorporation of patient perspectives [27]. Further, extant measures of patient engagement do not identify areas that can be targeted for intervention to improve engagement, but rather utilize downstream activities such as appointment attendance to signify engagement. Through our GCM methods incorporating perspectives of both patients and clinicians, we identify items that can be used to assess patient engagement efforts by healthcare systems. As a result, our study offers specific insight into areas that can be targeted for intervention by healthcare systems to improve patient engagement.

The concept map developed in this study advances the existing literature by providing a conceptualization of the dimensions of patient engagement. The five identified clusters include: Access, Attitudes and Behavior, Internal Resources, External Resources, and Relationship with Provider. Prior work on PE suffers from unsubstantiated definitions of PE, and high variability in how PE is conceptualized [28]. As a result, PE can be highly perspective or context dependent, and thus lacks reliability. Our concept map, developed via a modified GCM approach, addresses these deficits and offers a guide for researchers and practitioners aiming to measure PE.

Our findings show that Relationship with Provider is both the most important and most addressable dimension of patient engagement. Within this dimension, the go-zone plot calls attention to the importance of trust, feeling supported, confidence in care received, and feeling valued and respected, and each of these were rated highly addressable by both healthcare systems and patients. Each of these elements require intentional effort by healthcare systems and may be particularly salient for patients made vulnerable by their environment. For instance, in a population of HIV patients, Dang et al. showed that trust building is best begun early on in the clinician-patient relationship, and may include reassuring patients, telling them it’s ok to ask questions, showing and explaining their results, avoiding judgmental language and behaviors, and asking the patient about their treatments goals and preferences [29]. Similar findings have been reported for elderly patients [30]. Other identified trust-building activities include demonstrating competence, active listening, and providing explanations [31]. Supporting patients in these ways can also advance health equity and contribute to overcoming medical mistrust [32]. Participants in our study also noted these same elements were highly addressable by patients, suggesting that they influence provider choice and providing further encouragement for healthcare systems to focus on provider-patient relationships.

Our study elicits questions about how healthcare systems can support providers in engaging in these activities. Healthcare systems that prioritize the relationship between patients and providers, including developing trust, may be more oriented toward healthier outcomes in their communities [33, 34]. Healthcare systems might consider viewing trust as a process that begins before a patient enters a clinician’s office [35] — which includes marketing and communications from the healthcare system, and its community presence. This relationship has been shown to be particularly important with respect to vaccine uptake [36, 37], even prior to the COVID pandemic. One promising approach that has helped build relationships between healthcare systems and communities has been through community partnerships and coalition participation—an approach that not only reinforces positive interactions with healthcare system representatives, but also demonstrates ongoing community engagement [38–40].

Other healthcare system level interventions may help to address other dimensions of PE, such as Access or Internal or External Resources, pointing either towards health services accessibility in the form of appointment scheduling and reminders, or towards information about health issues and care in the form of educational materials and timely updates about patient health status. Both reminders and information, incidentally, are supported by the push to adopt patient portals and telemedicine to improve access to care [41]. While digital literacy may have historically been viewed as outside the scope of healthcare systems to address, these findings highlight the importance of digital tools to PE and suggest that healthcare systems should consider greater support for digital literacy interventions [42].

At the same time, the go-zone visualizations downplay the relevance of PE for some aspects traditionally considered in the literature important to address disparities due to differences in access to technology. Specifically, our findings show that elements like personal access to the internet and the patient’s level of digital literacy are difficult to address by the healthcare system, but are also relatively less important for PE, despite research showing how limited resources and capabilities can act as barriers to PE [43].

Our findings also highlight that aspects of a patient’s attitudes and behaviors are both important and factors that patients can address. One example is asking questions during a healthcare encounter. While this action is a patient behavior, healthcare systems can offer support to encourage patients to ask questions. Studies show the providing patients with tools such as the simple three-question AskShareKnow [44] approach can increase a patient’s perceived involvement in their care [45]. Likewise, the Agency for Healthcare Research and Quality developed a Question Builder application to help patients and caregivers prepare for appointments [46].

### Limitations

Our study findings should be interpreted in consideration of key limitations. To begin, our study sample — both the clinicians and patient advisors — may differ in their views on engagement from the general population for either group. Individuals who voluntarily responded to the statement generation or structuring steps could have higher standards related to patient engagement than non-respondents. Given our national recruitment approach, we do not have comparable data with which we could assess this bias. Further, we did not collect data regarding the demographic characteristics of participants, limiting our ability to examine the representativeness of our sample. Future work could sample more deliberately from specific sub-populations to identify differences in the PE concept map or the go-zones within those groups.

The temporal stability of our findings may also be questionable given drastic changes in healthcare due to the COVID-19 pandemic that emerged and evolved as our data was collected. Our GCM process took place between 2019 and 2021, a period that overlaps with the beginning of the pandemic in the U.S. in March 2020. It is possible, especially given the rapid transition to telehealth and virtual care options, that some elements (e.g., digital and health literacy) have increased in importance and even addressability as a result of the pandemic. Re-validation of our concept map would offer insight into how these dimensions might change over time in response to evolving contextual factors that within which healthcare systems operate.

## Conclusions

Healthcare systems lack meaningful approaches to supporting patient engagement, in part due to poor conceptual definition and measurement. Our findings advance the field by identifying the component dimensions of patient engagement. Further, our study nominates specific dimensions to target to increase patient engagement: improving relationships between providers and patients, supporting patients with external resources, and mechanisms that encourage positive patient attitudes and behaviors and increase access.

### Electronic supplementary material

Below is the link to the electronic supplementary material.

Supplementary Material 1

## Data Availability

The datasets used and/or analyzed during the current study are available from the corresponding author to all requesters who provide a methodologically sound proposal whose use has also been approved by an independent review committee. Interested parties will be required to complete an institutional Data Use Agreement.
