# Supplementary material for: Development of a conceptual model of the capacity for patients to engage in their health care: a group concept mapping study
Source: BMC Health Serv Res. 2023 Aug 10;23:846. doi: 10.1186/s12913-023-09785-x (PMC10413602; doi:10.1186/s12913-023-09785-x)
Supplement: Supplementary file 1 — Supplementary Material 1 [file 12913_2023_9785_MOESM1_ESM.pdf]

Additional file 1

ID #	Text	Bridging
1	Cluster name: Access	Avg 0.56
1	Able to get appointment	0.552
23	Insurance/Costs to patient	0.484
24	Internet access	0.503
37	Proximity to Health Care	0.615
44	Transportation	0.496
28	Medical Jargon	0.701
2	Cluster name: External resources	Avg 0.64
2	Able to contact provider outside of office	0.887
6	Appointment/Care Reminders	0.591
13	Educational Materials	0.664
14	Electronics	0.448
18	Fitness Classes	0.585
19	Health apps, fitness tech, wearables	0.475
25	Length of Visit	1.000
32	Patient Portal	0.502
3	Cluster name: Attitudes and behaviors	Avg 0.13
3	Patient advocates for self	0.062
7	Awareness of Treatment Options	0.215
8	Awareness of Treatment Plan	0.216
9	Communicate Symptoms to Provider	0.059
10	Patient is compliant	0.106
16	Empowered	0.213
20	Patient participates in health maintenance	0.072
30	Patient Asks Questions	0.126
34	Patient's positive attitude	0.059
35	Patient comes to appointment prepared	0.218
39	Resiliency	0.274
40	Patient self-efficacy/confidence	0.084

**47**	Patient is ready and motivated to participate in care	0.000
**4**	Cluster name: Internal resources	Avg 0.48
**5**	Anxiety	0.284
**21**	Health Status	0.584
**27**	Health Literacy	0.518
**31**	Patient Education Level	0.562
**42**	Stress	0.238
**43**	Support System	0.436
**26**	Digital Literacy	0.687
**29**	Understanding Healthcare System	0.553
**33**	Patient's Language	0.481
**5**	Cluster name: Relationship with provider	Avg 0.23
**4**	Provider advocates for patient	0.290
**11**	Confidence in care received	0.190
**12**	Culturally appropriate care	0.645
**15**	Empathy/Compassion	0.215
**17**	Feeling Supported	0.194
**22**	Included	0.133
**36**	Provider Listens	0.283
**38**	Rapport with Provider	0.137
**41**	Shared Decision-Making	0.231
**45**	Trust	0.100
**46**	Patient feels valued/respected	0.145
